# Native Magnetic Resonance T1-Mapping Identifies Diffuse Myocardial Injury in Hypothyroidism

**DOI:** 10.1371/journal.pone.0151266

**Published:** 2016-03-10

**Authors:** Xia Gao, Min Liu, Aijuan Qu, Zhe Chen, Yumei Jia, Ning Yang, Xiaomeng Feng, Jia Liu, Yuan Xu, Xinchun Yang, Guang Wang

**Affiliations:** 1 Department of Endocrinology, Beijing Chaoyang Hospital, Capital Medical University, Beijing, People's Republic of China; 2 Department of Radiology, Beijing Chaoyang Hospital, Capital Medical University, Beijing, People's Republic of China; 3 Department of Physiology and Pathophysiology, School of Basic Medical Science, Capital Medical University, Beijing, People's Republic of China; 4 Heart Center, Beijing Chaoyang Hospital, Capital Medical University, Beijing, People's Republic of China; Fondazione G. Monasterio, ITALY

## Abstract

**Background and Aim:**

Hypothyroidism (HT) is characterized by thyroid hormone deficiencies, which can lead to diffuse myocardial interstitium lesions in patients with HT. Myocardial longitudinal relaxation time (T1) mapping is a potential diagnostic tool for quantifying diffuse myocardial injury. This study aimed to assess the usefulness of T1 mapping in identifying myocardial involvement in HT, and determine the relationship between T1 values and myocardial function.

**Methods:**

A cross-sectional study was conducted with 30 untreated HT patients alongside 23 age- and sex-matched healthy controls. All subjects underwent cardiac magnetic resonance (CMR) with non-contrast (native) T1 mapping using a modified Look-Locker inversion-recovery (MOLLI) sequence to assess the native T1 values of myocardium and cardiac function.

**Results:**

Native myocardial T1 values were significantly increased in HT patients, especially those with pericardial effusion (*p* < 0.05), compared with healthy controls. In addition, significantly reduced peak filling rate (PFR) and prolonged peak filling time (PFT) were obtained (*p* < 0.05) in HT patients compared with controls. Furthermore, stroke volume (SV) and cardiac index (CI) were significantly lower in HT patients than controls (all *p* < 0.05). Interestingly, native T1 values were negatively correlated with free triiodothyronine (FT3), PFR, SV and CI (all *p* < 0.05).

**Conclusion:**

Diffuse myocardial injuries are common in HT patients, and increased T1 values are correlated with FT3 and cardiac function impairment. These findings indicate that T1 mapping might be useful in evaluating myocardial injuries in HT patients.

## Introduction

Hypothyroidism (HT) is caused by reduced production or inadequate activity of thyroid hormones. The prevalence of overt HT in the general population varies from 0.1 to 3.7%, with women having a greater risk than men [1, 2]. The cardiovascular system has long been recognized as one of the most important targets of thyroid hormones [3]. Changes of cardiac structure and function depend on the degree and duration of thyroid hormone deficiency in HT patients [4, 5]. A growing body of clinical evidence suggests that HT is associated with increased cardiovascular risk and mortality [5–7]. Even acute HT was associated with left ventricular dysfunction [8]. Chronic thyroid hormone deficiencies can result in profound changes in cardiac function regulation and cardiovascular hemodynamics, such as prolonged systolic and early diastolic times, decreased cardiac preload due to impaired diastolic function as well as increased cardiac afterload and reduced chronotropic and inotropic functions [9]. Early detection and assessment of myocardial involvement is crucial for HT patients.

Myocardial interstitium is essential for normal structure integrity and mechanical functions, which is under the regulatory influence of thyroid hormones [10, 11]. HT is characterized by diffuse interstitial space expansion with increased extracellular collagen, normally through the development of fibrosis, and accumulation of mucopolysaccharide substances [12, 13], which have an important role in the regulation of tissue hydration. Animal and clinical studies have shown that myocardial fibrosis is associated with abnormal cardiac remodeling, increased ventricular stiffness and worsened ventricular function [14–16]. In recent clinical studies, fibrosis has also been recognized as an independent predictive factor of adverse cardiac outcome such as cardiac death, unstable angina, and heart failure [17–19]. The only methodology for myocardial fibrosis evaluation previously available is the histopathological assessment of endomyocardial tissue biopsies, which was limited by its invasive nature and sampling error. More importantly, biopsy can't provide information on the extent of ventricular involvement [20]. Over the past decades, cardiac magnetic resonance (CMR) using late gadolinium enhancement (LGE) imaging technique can non-invasively detect the patterns and distribution of regional (typically replacement) fibrosis and scar. Based on the differences of gadolinium distribution between healthy and diseased myocardia, the conventional LGE technique has been used to detect regional fibrosis in this tissue [21]. However, this method is not suitable for diffuse myocardial fibrosis because of the reduced amounts of normal non-fibrotic myocardium available to compare with affected areas.

Myocardial longitudinal relaxation time (T1) mapping of the myocardium is a parametric reconstructed image, where each pixel’s intensity directly reflects the T1 relaxation time of the corresponding myocardial voxel. Therefore, the T1 value provides an intrinsic signal from both the interstitium and myocytes. In addition, T1 mapping is now considered an emerging technique for assessing diffuse myocardial interstitial fibrosis in the whole heart, truly reflecting the global myocardial fibrosis burden; furthermore, T1 mapping is highly sensitive to myocardial water and superior to conventional T2-weighted CMR in detecting myocardial oedema [22, 23], this capability is important for overt HT patient. A previous study showed the high sensitivity and specificity of T1 mapping for differentiating patients with diffuse myocardial fibrosis and normal volunteers [24]. In addition, increased native T1 values are known to correlate well with histology in diffuse fibrosis [11].

One of the distinctive factors of HT cardiomyopathy pathology is diffuse interstitial space expansion, which normally occurs through the development of fibrosis. Overt HT also can lead to myocardial oedema due to the extensive deposits of acid mucopolysaccharide [25]. T1 mapping is a potential useful toll in assessing HT patients with diffuse fibrosis and oedema. In addition, T1 mapping enables direct quantification (in milliseconds) of myocardial signal on a standardized scale [26]. However, the study on global myocardial interstitium injury in HT has not been reported.

In the present study, we therefore hypothesized that T1 mapping might assess diffuse myocardial interstitium injury quantitatively in HT patients. In addition, we aimed to investigate that T1 value is a potential index, which is related to myocardial dysfunction of HT patients.

## Patients and Methods

### Subjects

A total of 30 female patients, with overt HT caused by chronic lymphocytic thyroiditis, free from concomitant disease and without medical treatment, were assessed in this cross-sectional study. Inclusion criteria were age between 18 and 49, decreased free thyroxine (FT4) level (≤0.4 ng/dl), increased serum thyroid stimulating hormone (TSH) level (≥100 μIU/ml), and positive anti-thyroid peroxidase antibody. Patients with known heart disease (previous myocarditis, myocardial infarction, arrhythmia, heart failure and other chronic cardiac condition), diabetes, hypertension, kidney failure, asthma, chronic obstructive pulmonary disease, neoplastic disease, pregnancy, claustrophobia and metal implants, were excluded. Patients were compared to 23 healthy age-matched female controls. For all subjects, sex, age, height, body weight, heart rate and blood pressure were recorded, and body surface area (BSA) was derived as BSA = 0.007184×height (cm)^0.725^×weight (kg)^0.425^. The study was conducted from March to October 2014 at the Department of Endocrinology in Beijing Chao-yang Hospital. The protocol was designed according to Declaration of Helsinki guidelines and approved by the Medical Ethics Committee of Beijing Chaoyang Hospital. Written informed consent was obtained from all patients.

### Measurements

Blood samples were taken in the morning after an overnight fast and collected from the antecubital vein. FT3, FT4, TSH, cardiac troponin I (cTNI) and creatinine levels were measured by electrochemiluminescence immunoassay (ECLIA), high-sensitivity C-reactive protein (hsCRP) level was measured by nephelometry immunoassay. All the blood variables were measured using an Abbott Architect i2000 (Abbott Diagnostics, Abbott Park, IL, USA). Reference intervals for FT3, FT4, TSH, cTNI, hsCRP and creatinine were 1.71–3.71 pg/ml, 0.7–1.48 ng/dl, 0.35–4.94 μIU/ml, 0–0.09ng/ml, 0–3mg/L, and 53.0–115.0 μmol/l respectively.

CMR studies were performed with the patient supine, using clinical 3 T scanners on a Tim Trio System (Siemens Healthcare, Erlangen, Germany) and a 32-channel phased-array chest coil used for data acquisition. After location, cine images were acquired by gapless whole heart coverage of short-axis slices. Then complete stack of short axis images were obtained during a gentle expiratory breath hold and cardiac gating for TSE-T2-weighted imaging (TSE-T2WI) with fat suppression [27] and native T1 mapping with Modified Look-Locker inversion-recovery (MOLLI) sequence without contrast administration [28]. LGE imaging was performed after T2WI and native-MOLLI as previously decribed [29]. LGE imaging was acquired in the short-axis planes using a T1-weighted phase-sensitive inversion recovery (PSIR) sequence 10 minutes after intravenous administration of the contrast agent (Gadopentetate dimeglumine-Gd-DTPA, Magnevist, Bayer Healthcare; 0.20 mmol/kg body weight). Slice thichness for cine, T2-weighted and T1-mapping images was 8 mm. All routine CMR images and maps were analyzed with Argus (SYNGO MMWP Workstation, Siemens AG).

Left ventricular (LV) function was determined with the Argus software according to the Society for CMR guidelines for reporting CMR examinations [17]. LV endocardial and epicardial borders were manually contoured at end-systole and end-diastole. LV end-diastolic volume (LVEDV) and end-systolic volume (LVESV) were determined using the Simpson’s rule. Ejection fraction (EF) was derived as EF = (LVEDV-LVESV)/LVEDV. Myocardial mass was calculated by subtracting the endocardial volume from the epicardial volume, based on known knowledge of myocardial specific gravity (1.05g/cm^3^). All volumetric indexes were normalized to BSA.

T1 relaxation maps were analyzed as previously described [28]. Briefly, after T1 maps were obtained, short axis images were automatically contoured to outline the endocardium and epicardium. Previous studies showed substantial segmental T1 variation, which was greatest in lateral and smallest in septal segments [30, 31]. Considering the analysis of T1 values in four segments might provide more information on myocardial lesions, so the region of interest (ROI) was manually drawn in the anterior, septal, inferior, and lateral segments of the left ventricle on the mid-short axial slices to quantify T1 values.

### CMR image quality control

Shimming and center frequency adjustments were performed to generate off-resonance artifact free images. Quantitative image analyzes for T1 mapping were performed by 2 expert CMR cardiologists. Intraobserver agreement for T1 measurements was assessed by blinded repeat analysis of images one month after the initial analysis by cardiologist 1.

### Statistical analysis

Data analysis was performed with SPSS Statistics, version 21.0 (SPSS, Chicago, Illinois, USA) and MedCalc 15.10 (MedCalc Software, Mariakerke, Belgium). Normality of data was tested using the Kolmogorov-Smirnov test. Normally distributed data are mean±standard deviation (SD); non-parametric data are presented as median with interquartile range (IQR).The differences between the two groups were analyzed by independent Student’s t-test for unpaired samples, or the Mann-Whitney U test for non-parametric data; one-way analysis of variance (ANOVA) followed by Tukey’s post hoc test was used for multiple comparisons of group means. Intra- and interobserver agreement was assessed by coefficients and Bland-Altman plots. Spearman or Pearson analysis was used to assess correlations. P<0.05 was considered statistically significant.

## Results

### Patients’ baseline characteristics

Baseline characteristics for all patients are summarized in **Table 1**. No differences in age, sex, heart rate (HR), BSA and blood pressure (BP) were found between the HT and control groups (all *p*>0.05). However, BMI was higher in the HT group compared with controls (26.00±3.70 vs. 22.02±3.28 kg/m^2^, *p* = 0.0002). In addition, plasma FT3 levels were significantly lower in HT individuals compared with the control group [1.65(1.16–2.12) vs. 2.71(2.58–3.13) mmol/l, *p*<0.0001), indicating a severe disease state in these patients. The median level of hsCRP in the HT group was significantly higher than that in the control group [0.64 (0.05–1.46) vs. 0 (0–0) mg/L, *p* = 0.0013]. However, there was no significant difference for the median cTNI level between the two groups. Finally, creatinine levels were higher in HT patients compared with the control group (76.26±12.21 vs. 60.82±11.69 μmol/l, *p*<0.0001), but remained within the normal range for all study subjects.

**Table 1:** Baseline Characteristics of the Control and Hypothyroidism Groups. Summary of the clinical characteristics and laboratory results of the study participants in 23 controls and 30 patients with hypothyroidism. Data were expressed as the mean±SD or median (interquartile range). Abbreviations: BMI, body mass index; BP, blood pressure; BSA: body surface area; cTNI: cardiac troponin I; FT3, free triiodothyronine; FT4, free thyroxine; HR, heart rate; hsCRP: high-sensitivity C-reactive protein; TSH, thyroid stimulating hormone.

	Controls (n = 23)	Hypothyroidism (n = 30)	p value
Female (n)	23	30	-
Age (years)	35.43±8.25	36.60±7.87	0.603
SBP (mmHg)	116.7±9.93	119.1±12.65	0.442
DBP (mmHg)	68.39±6.93	71.73±9.03	0.147
BMI (kg/m^2^)	22.02±3.28	26.00±3.70	***0*.*0002***
BSA (m^2^)	1.66±0.17	1.74±0.14	0.073
HR (beats/min)	69.30±9.18	68.43±9.65	0.741
FT3 (pg/ml)	2.71 (2.58–3.13)	1.65 (1.16–2.12)	***<0*.*0001***
FT4 (ng/dl)	1.10±0.14	<0.4	-
TSH (μIU/ml)	2.37±1.08	>100	-
hsCRP (mg/L)	0 (0–0)	0.64 (0.05–1.46)	***0*.*0013***
cTNI (ng/dl)	0 (0–0)	0 (0–0.025)	0.310
Creatinine (μmol/l)	60.82±11.69	76.26±12.21	***<0*.*0001***

### Cardiovascular Magnetic Resonance

Pericardial effusions were detected in 12 HT patients as shown in **Table 2**, which also summarizes CMR parameters. Similar values were obtained for LV mass, EF and ESV in both groups (all *p*>0.05). In addition, no statistically significant differences were obtained in peak ejection time (PET, 130.60±29.41 vs. 145.50±31.58 ms, *p* = 0.087) and peak ejection rate (PER, 3.55±0.52 vs. 3.45±0.85 EDV/s, *p* = 0.629), between the control and HT groups. EDV values in HT patients were slightly lower compared with controls (51.26±11.30 vs. 56.71±10.18, *p* = 0.075), but the difference did not reach statistical significance. However, prolonged peak filling time (PFT, 154.80±48.33 vs. 129.8±31.71 ms, *p*<0.05) and decreased peak filling rate (PFR, 3.59±0.84 vs. 4.27±0.99 EDV/s, *p*<0.05) were significantly altered in the HT group compared with control values, indicating an impaired diastolic function in HT patients. In addition, stroke volume (SV, 30.51±7.42 vs. 35.50±5.95 ml/m^2^) and cardiac index (CI, 2.07±0.54 vs. 2.43±0.33 l/min/m^2^, *p*<0.05) were significantly reduced in the HT group compared with controls, suggesting a poor cardiac output in HT individuals.

**Table 2:** Cardiovascular Magnetic Resonance Parameters of the Control and Hypothyroidism Groups. Abbreviations: CI, cardiac index; EDV, end diastolic volume; EF, ejection fraction; ESV, end systolic volume; IVS, interventricular septum; LVAW, left-ventricular anterior wall; LVIW, left-ventricular inferior wall; LVLW, left-ventricular lateral wall. PER, peak ejection rate; PET, peak ejection time; PFR, peak filling rate; PFT, peak filling time; SV, stroke volume.

	Controls (n = 23)	Hypothyroid (n = 30)	p value
EF (%)	63.01±6.09	59.59±8.95	0.122
PET (ms)	130.6±29.41	145.5±31.58	0.087
PFT (ms)	129.8±31.71	154.80±48.33	***0*.*040***
EDV (ml/m^2^)	56.71±10.18	51.26±11.30	0.075
ESV (ml/m^2^)	21.25±5.93	20.80±6.58	0.798
SV (ml/m^2^)	35.50±5.95	30.51±7.42	***0*.*011***
CI (l/min/m^2^)	2.43±0.33	2.07±0.54	***0*.*007***
Mass (g/ m^2^)	50.18±10.59	52.42±14.34	0.533
PER (EDV/S)	3.55±0.52	3.45±0.85	0.629
PFR (EDV/s)	4.27±0.99	3.59±0.84	***0*.*010***
T1-LVAW (ms)	1083±51.28	1220±75.85	***<0*.*001***
T1-IVS (ms)	1048±66.29	1175±81.87	***<0*.*001***
T1-LVIW (ms)	1062±56.56	1179±80.21	***<0*.*001***
T1-LVLW (ms)	1066±47.69	1185±81.79	***<0*.*001***
Pericardial effusion (n)	0	12	**-**

### T2-weighted CMR, native myocardial T1 and LGE imaging

**Fig 1:**
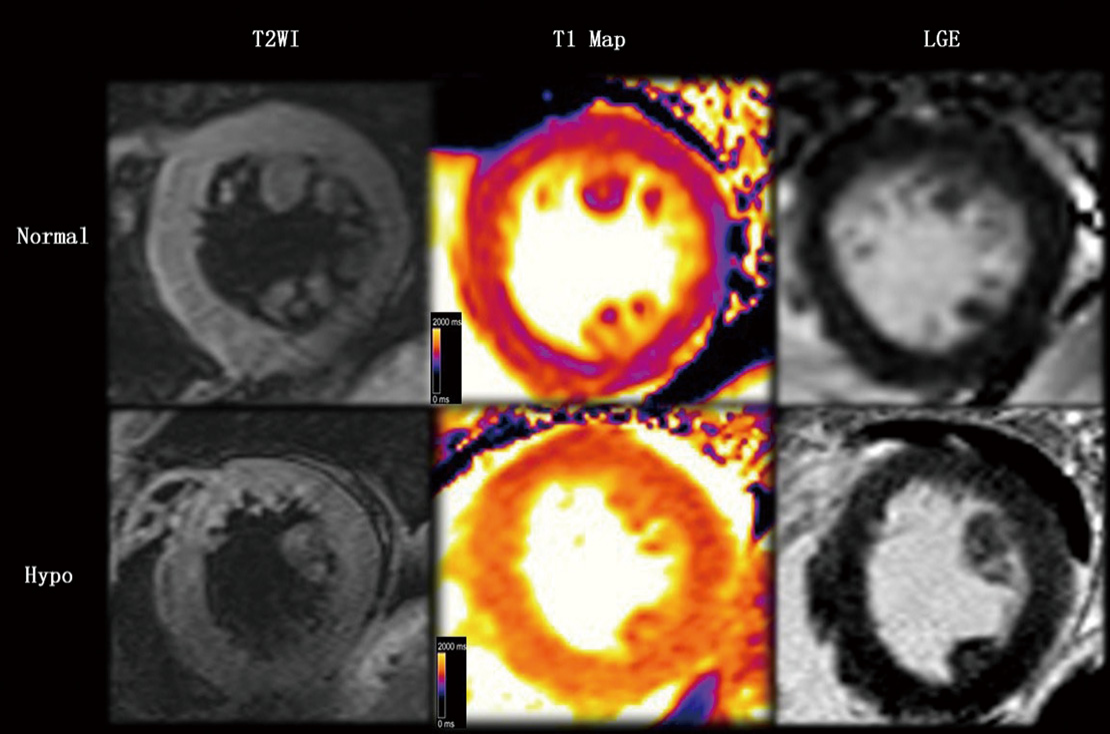
Representative cardiac magnetic resonance images from a 42-year-old patient with overt hypothyroidism (Hypo) and normal control (Normal). (**Left**) T2WI showed a homogenous intensity within the left ventricular and overt pericardial effusion in Hypo. (**Middle**) color T1 maps based on a native modified Look-Locker inversion. Note the markedly elevated myocardial T1 time in the Hypo (T1 = 1301 ms, orange range of the color scale) compared with the Normal control (T1 = 1040 ms, purple range of the scale). (**Right**) on LGE, myocardial had a homogenous intensity in Hypo which was similar to intensity in Normal control.

**Fig 2:**
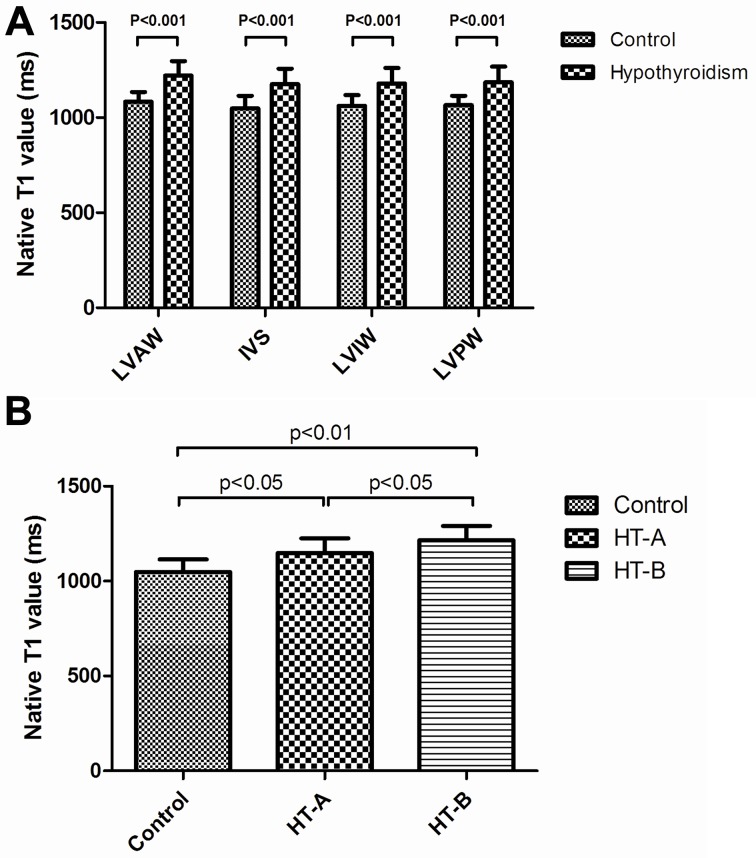
Native T1 values in controls and HT patients with or without pericardial effusion. **A:** Native T1 values within four segments of the left ventricle comparing the euthyroid control to overt hypothyroidism group. **B:** Native T1 values within ventricular septum comparing the euthyroid control to overt hypothyroidism groups with (HT-B) or without (HT-A) pericardial effusion.

On conventional T2WI, HT patients had a homogenous intensity within the LV and overt pericardial effusion (**Fig 1**). Native MOLLI (**Table 2** and **Figs 1 and 2A)** showed significantly higher native myocardial T1 values in HT patients compared with healthy controls. Interestingly, T1 values within interventricular septum were even higher in the severe HT subgroup with pericardial effusion (1216±74.97 ms) compared with HT individuals without pericardial effusion (1148±76.77 ms) (**Fig 2B)**. On LGE, myocardial also had a homogenous intensity in HT patients which was similar to intensity in normal controls (**Fig 1**).

### T1 mapping reproducibility

**Fig 3:**
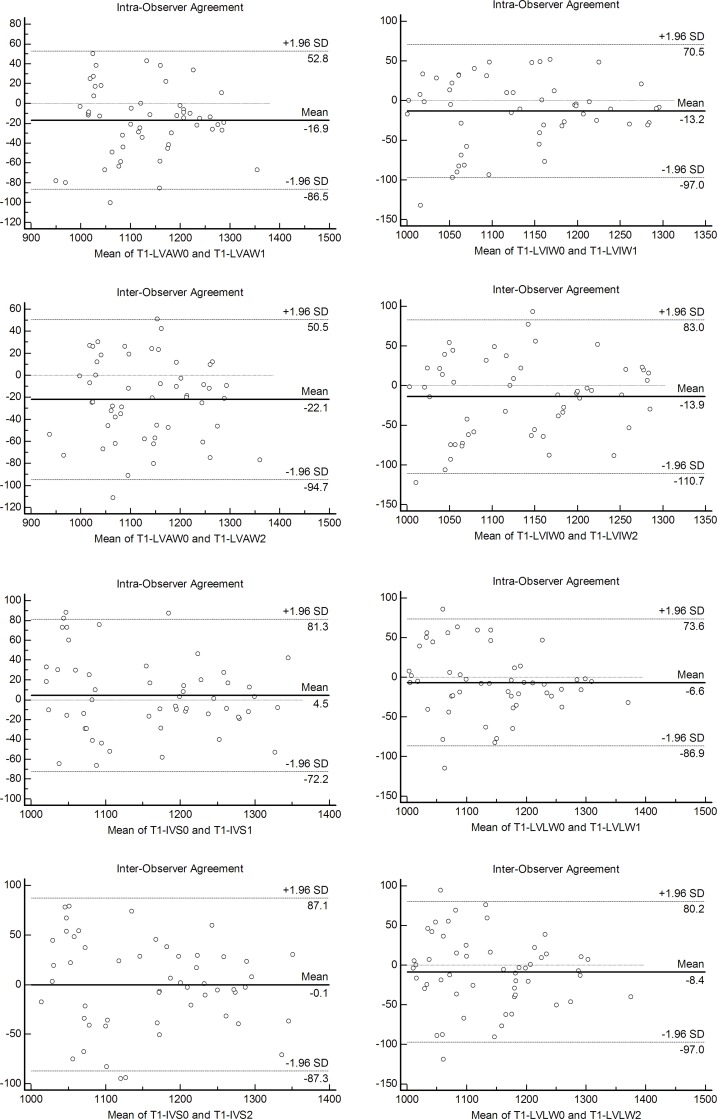
The intra-observer and inter-observer Bland-Altman plot for native T1 values within the LVAW, IVS, LVIW and LVLW. Bland-Altman plots demonstrating the intra-observer (LVAW0 and LVAW1, IVS0 and IVS1, LVIW0 and LVIW1, LVLW0 and LVLW1) and inter-observer (LVAW0 and LVAW2, IVS0 and IVS2, LVIW0 and LVIW2, LVLW0 and LVLW2) agreement for T1 measurements. Horizontal solid lines represent mean differences, and dashed lines 95% limits of agreement. IVS: interventricular septum; LVAW: left-ventricular anterior wall; LVIW: left-ventricular inferior wall; LVLW: left-ventricular lateral wall; SD: standard deviation.

There was excellent intra- and inter-observer correlation for native T1 values within LVAW, IVS, LVIW and LVLW (intra-observer coefficients: r = 0.94, 0.92, 0.89 and 0.91, respectively; inter-observer coefficients: r = 0.93, 0.90, 0.85 and 0.88, respectively). In addition, intra- and inter-observer Bland-Altman plots for native T1 values within LVAW, IVS, LVIW and LVLW showed good agreement (**Fig 3**).

### Correlation between myocardial native T1 value and parameters of LV diastolic and systolic function

**Fig 4:**
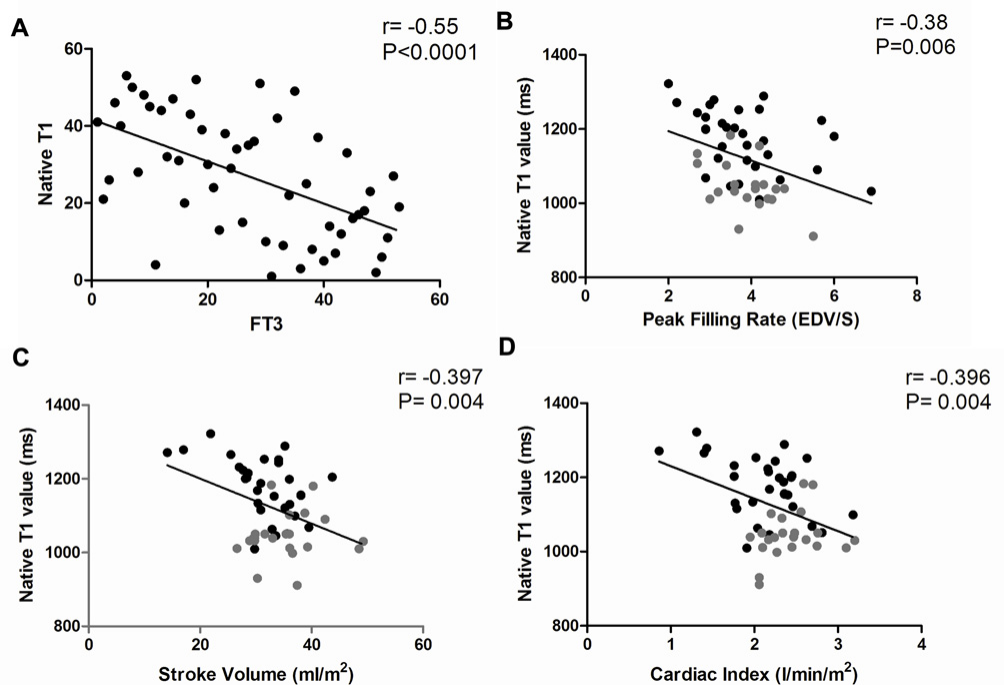
Bivariate analysis of the correlation between T1 value within interventricular septum and FT3, Peak Filling Rate, Stroke Volume and Cardiac Index in controls and patients with HT. Spearman or Pearson analysis was used to assess the correlation between T1 value and FT3 (**A**), Peak Filling Rate (**B**), Stroke Volume (**C**) and Cardiac Index (**D**). The central line represents the regression line. Black dot: HT patients; Gray dot: Controls.

We next assessed whether myocardial damage, as reflected by increased T1 values within IVS, is related to LV diastolic or systolic function. Interestingly, a significant negative correlation was obtained between the myocardial native T1 value and plasma FT3 levels (Spearman correlation, r = -0.55, *p*<0.0001) (**Fig 4A**). In addition, the native T1 value was inversely correlated with PFR (r = -0.38, p = 0.006) (**Fig 4B)**, SV (r = -0.397, p = 0.004) (**Fig 4C)** and CI (r = -0.396, p = 0.004) (**Fig 4D).** These findings suggest that diffuse myocardial lesions are associated with impaired LV diastolic and cardiac output. Therefore, T1 mapping can be considered a novel noninvasive tool for evaluating myocardial lesions and cardiac function in patients with HT.

## Discussion

In this study, we demonstrated that native myocardial T1 values were significantly increased in HT patients, especially those with pericardial effusion, compared with healthy controls. However, T2WI and LGE were limited to detect the diffuse myocardial injury in HT. Furthermore, reduced PFR and prolonged PFT were obtained in HT patients compared with controls. However, SV and CI were significantly lower in HT patients than in controls. Interestingly, native T1 values were negatively correlated with FT3, PFR, SV and CI, which may contribute to cardiac dysfunction in HT patients. These results indicated that CMR, particularly T1 mapping, can be used for the detection and quantitative assessment of myocardial involvement in HT patients.

As shown above, HT patients had significantly increased native T1 values, especially those with pericardial effusion. In the absence of other causes of interstitium damage such as myocardial infarction, myocarditis or amyloid, increased T1 values are regarded as non-invasive surrogate of diffuse myocardial fibrosis and oedema induced by HT. LGE-CMR relies on the delivery of intravenous gadolinium chelate to the myocardium, which is a biologically inert tracer that freely distributes in extracellular space but does not cross the intact cell membrane. So LGE can be used to detect the regional increase of extracellular volume caused by fibrosis. Contrast relies on signal intensity differences between normal myocardium and fibrosis [32]. However, as shown in our study, no apparent areas of LGE were found in HT patients on LGE MRI, it may be impossible to define an area of clearly unaffected myocardium as a “nulled” reference. Similarly, T2WI is sensitive to regional as well as global increases in myocardial water content. Identification of oedema depends on the signal differences between affected myocardium and remote normal myocardium or should be verified by calculating the ratio between myocardium and skeletal muscle [33]. However, in cases of overt HT, the skeletal muscle was also affected [34], and T2WI showed a homogenous intensity within the left ventricular. Thus, T1 mapping is a potential additional quantitative tool for detection of cardiac involvement in HT patients. As both fibrosis and oedema, which likely coexist in HT patients [25], would increase native T1 values, it may be challenging to distinguish these two pathological changes based on imaging alone.

The T1 mapping sequence used in this study was the MOLLI sequence [28], which provides high-resolution native T1 maps of human myocardium within a single breath-hold. Meanwhile, MOLLI allows a highly reproducible T1 map with high levels of inter and intra-observer agreement [35]. Thanks to these advantages, the MOLLI technique overcomes the limitations of prolonged acquisition time and motion. In addition, native T1 mapping does not require the use of an exogenous contrast agent, an additional advantage for subjects with significant renal impairment, as found in some HT patients, due to marked reduction in renal flow.

T4 is not transported into the heart, so the cardiac phenotype is extremely sensitive to changes in serum T3. As shown above, a negative correlation between T1 values and FT3 was demonstrated. These data indicated that decreased thyroid hormone concentrations in the blood might be related to the myocardial interstitium lesions, in line with previous findings [36]. In addition, myocardial interstitium lesions were correlated with the extent of thyroid hormone deficiency.

Interestingly, BMI in HT patients was significantly higher than control values. This has limited importance, however, as all cardiac volume data were normalized to BSA. Severe HT patients exhibited significantly reduced PFR and prolonged PFT in this study, reflecting an overt effect of thyroid hormone deficiency on the heart [5]. These findings strongly suggested impaired diastolic filling and reduced myocardial relaxation in patients with severe HT. Finally, cardiac preload was reduced due to the impaired diastolic function and decreased blood volume. During this process, myocardial interstitium damage may not be ignored [37]. In addition, cardiac output in HT patients was abnormal in our study, with significantly lower SV and CI values, corroborating a previous study [38]. Because there was no difference in HR between the HT and control groups, the decreased SV and CI were partially driven by the decreased preload and increased afterload [39]. Early hypothyroidism myocardial lesions can induce abnormal diastolic function accompanied with progressive systolic dysfunction [38, 40]. Patients' EF values were normal, and EDV in patients with hypothyroidism more pronouncedly decreased compared with ESV in the present study, indicating no overt systolic dysfunction; decreased SV and CI were mainly related to abnormal diastolic dysfunction.

In the present study, T1 value was shown to correlate with cardiac dysfunction. To our knowledge, this is the first study describing the association of myocardial damage and LV function in HT patients. It was previously demonstrated that myocardial fibrosis significantly contributes to the pathogenesis of myocardial relaxation abnormalities [41]. Our findings support the hypothesis that diffuse myocardial interstitium injury may lead to impaired myocardial function.

Some limitations of this study should be mentioned. It had a cross-sectional design, and confounding factors, such as unknown comorbidities, may not be evenly distributed in both groups. In addition, the study was carried out in a single center with a relatively low sample size. Given that previous reports revealed elevated hs-CRP and cTNI levels in patients with hypothyroidism [42–44], while myocarditis or myocardial necrosis can also lead to increased T1 values [45], we hypothesized that there may be correlations between T1 values and hsCRP and cTNI levels. However, we found no significant association of T1 value with hsCRP or cTNI level (data not shown). These results might be due to the small sample size in this study, and further studies are needed to verify these findings. Furthermore, the correlation between FT3 and T1 value is not significant in HT group, which might be related the small size of patients. Finally, native myocardial T1 reflects a composite signal from both the intracellular and the extracellular compartment. Extracellular volume fraction (ECV) measures the extracellular space, in the absence of myocardial oedema, expansion of the myocardial collagen volume fraction is responsible for most of the extracellular matrix expansion [46], but myocardial oedema can be induced by HT. Therefore, further studies (including after correction of HT) are warranted to confirm our findings.

In conclusion, myocardial involvement is common in patients with overt HT, as measured by native T1 mapping. This new CMR sequence is capable of identifying diffuse myocardial injury not readily recognized by T2WI and LGE. In the cases of HT, increased T1 value can be regarded as an index of diffuse myocardial injury most likely caused by fibrosis and oedema, correlating with serum FT3 level as well as diastolic function impairment and reduced cardiac output. T1 mapping furthers our understanding of the changes to the ECM, which plays an important role in the pathogenesis of HT. Finally, CMR, particularly T1 quantification, is an optimal tool for detection of myocardial involvement for clinical use in HT patients.

## Supporting Information

S1 Checklist PLOS ONE clinical studies checklist. (DOCX)

S2 Checklist SROBE checklist. (DOCX)
